# Acute acquired comitant esotropia in the smartphone era: a narrative review

**DOI:** 10.1007/s10384-026-01417-1

**Published:** 2026-09-16

**Authors:** Eizo Tanaka, Yuki Morizane, Seiko Fujii, Mari Yaida, Weiying Sun, Satoshi Hasebe

**Affiliations:** 1 Department of Ophthalmology, Graduate School of Medicine, Dentistry and Pharmaceutical Sciences, Okayama University, 2-5-1 Shikata-cho, Kita-ku, Okayama, 700-8558 Japan; 2 Department of Ophthalmology 2, Kawasaki Medical School, 2-6-1 Nakasange, Kita-ku, Okayama, 700-8505 Japan

**Keywords:** Acute acquired comitant esotropia, Smartphone, Digital display, Near work, Vergence adaptation

## Abstract

**Purpose:**

Acute acquired comitant esotropia (AACE) has received increasing attention in the smartphone era, following numerous clinical reports suggesting an association with intensive and prolonged device use. However, it remains unclear whether smartphone use is a direct cause of AACE, a trigger in susceptible individuals, or a surrogate marker for broader modern lifestyle changes that may contribute to its pathogenesis.

**Study design:**

Focused narrative review.

**Methods:**

This article presents a focused narrative review of the literature on AACE in relation to intensive smartphone or other handheld digital-display use, with an emphasis on clinical evidence, exposure characteristics, potential sources of bias, and biologically plausible mechanisms.

**Results:**

Most of the available evidence comes from retrospective studies and uncontrolled visual hygiene trials, both of which are often limited by selection bias and inaccurate self-reporting of screen time. In addition, publication bias is a major concern; cases mentioning smartphone use may be more likely to be published than otherwise similar cases without reported smartphone exposure.

**Conclusions:**

Prolonged near-viewing on handheld digital displays is a biologically plausible contributing factor to AACE in some patients, particularly through sustained high vergence demand at short viewing distances. Nevertheless, current data are insufficient to establish a population-level causal relationship. Future studies should incorporate objective exposure metrics, standardized phenotyping, and explicit adjustment for age, refractive error, and objectively assessed near-work load to clarify the strength and nature of this association.

## Introduction

Acute acquired comitant esotropia (AACE) is characterized by the acute onset of esodeviation, uncrossed diplopia, and full ocular motility. It is not a new clinical entity; rather, it has long been recognized as a form of acute convergent strabismus in otherwise healthy individuals. Historically, AACE was considered uncommon in everyday clinical practice [1, 2]. Nevertheless, the last decade has changed this perspective. We have seen a surge in publications indicating an association between AACE and the intensive use of smartphones and other handheld digital displays, to the point where the term smartphone-induced strabismus is now employed in public discourse [2–9].

Currently, a notable discrepancy exists in the available evidence: although smartphones are ubiquitous, with billions of users globally [10], AACE remains relatively uncommon. If smartphone use were a direct cause of AACE, a substantial worldwide increase in cases would be expected. Instead, the actual data on case trends vary significantly from one study to another [11, 12]. These inconsistencies warrant closer examination. The question is not just whether intensive and prolonged near viewing can trigger a breakdown in eye alignment in certain people, but rather why it happens to some and not others, and what other factors might be at play.

This review examines the clinical evidence, exposure characteristics, potential biases and confounders, and biologically plausible mechanisms linking intensive and prolonged handheld digital-display use to AACE.

## Methods

### Search strategy and study selection

We conducted a focused narrative review. A targeted search was performed across PubMed, Web of Science, and Scopus on November 1, 2025, using combinations of the following terms: acute acquired comitant esotropia, AACE, smartphone, digital display, tablet, and near work. The search was restricted to publications directly relevant to the pathogenesis of AACE in the context of handheld digital-display use and associated near-viewing behaviors. We included case reports, case series, retrospective observational studies, registry-based studies, and interventional studies. From this search and reference checking, eight representative clinical publications were selected for tabulation because they address key aspects of handheld digital-display exposure, viewing distance, risk factors, time trends, registry data, or visual hygiene interventions. Additional relevant case reports and small case series are cited in the text.

## Clinical evidence and its limitations

### Case reports and case series: plausible temporality, weak causal inference

The early evidence on smartphone-induced AACE primarily comprises descriptive case reports and small clinical series. An initial case report documents acute-onset comitant esotropia in an adolescent with intensive smartphone usage, noting partial resolution following a reduction in near-work load [4]. Subsequently, a retrospective series of 12 adolescents identifies AACE in individuals exceeding four hours of daily smartphone use for several months; notably, behavioral modification led to a reduction in the angle of deviation in multiple cases, although surgical intervention remained necessary for others [3]. Similarly, other small cohorts report acute uncrossed diplopia and esotropia in pediatric populations with excessive device use, proposing accommodative spasm as a potential pathophysiological mechanism [5, 6]. During the COVID-19 pandemic, additional series identified clusters of AACE in children participating in prolonged smartphone-based remote learning, further speculating that intensive near viewing serves as a precipitating factor [6, 8]. Key studies investigating the association between AACE and handheld digital-display use [3, 7–9, 11, 13–15] are summarized in Table 1. While the association is suggestive, the high risk of bias inherent in retrospective, self-reported data must be carefully considered [16].

**Table 1:** Summary of key studies investigating the link between AACE and handheld digital-display use [3, 7–9, 11, 13–15]

Study (year)	Country	Study design	N	Age range	Exposure criteria	Key findings
Lee et al. (2016)	Korea	Case series	12	7–16 yrs	≥4 h/day smartphone use	Large esodeviation; decreased after smartphone restriction in 9/12 patients.
Topcu Yilmaz et al. (2020)	Turkey	Retrospective series	27	Children	Intensive DD use, near work	ACE was associated with intensive digital-display near work.
Neena et al. (2022)	India	Retrospective series	15	Children	Increased near work, often involving smartphones	AACE followed increased near work; some cases required surgery.
Van Hoolst et al. (2022)	Belgium	Retrospective case-control	10	5–15 yrs	Tablet/smartphone viewing distance	AACE cases had shorter tablet/smartphone viewing distances than controls.
Cai et al. (2023)	China	Retrospective case-control	83	5–52 yrs	Near work, spectacles, posture	No spectacles and supine near work were independent risk factors.
Okita et al. (2023)	Japan	Single-center time trend	171	<30 yrs	AACE trend and excessive smartphone use	AACE increased over 12 years, especially with excessive smartphone use.
Iimori et al. (2023)	Japan	Multicenter registry	194	5–35 yrs	Daily DD use	Phenotype differed by age; children had larger angles despite shorter use.
Iimori et al. (2025)	Japan	Non-randomized intervention	181	5–35 yrs	Reduced handheld DD use	Angle decreased only in the high-use DD group after 3 months.

N indicates the number of AACE/ACE

*AACE* acute acquired comitant esotropia, *ACE* acquired comitant esotropia, *DD* digital display

These reports yield three recurring clinical observations: (1) a distinct temporal association between intensified near viewing and the onset of symptoms, (2) a frequent presentation of comitant esodeviation with moderate-to-large angles, and (3) sporadic improvement following visual hygiene interventions (e.g., reduced usage and increased viewing distance). Nevertheless, temporal proximity alone is insufficient to establish a causal relationship, and these study designs are particularly susceptible to selective reporting and confounding variables (discussed in Section "Bias, confounding, and the “smartphone as a convenient villain”").

### Comparative observational studies: a signal for short working distance, but not definitive

A notable case-control study utilized questionnaire-based assessments to compare children with presumed digital display-induced AACE against age-matched controls. The affected cohort exhibited significantly shorter working distances during device use compared to the control group. Furthermore, while diplopia improved in some patients following behavioral hygiene interventions, a substantial proportion ultimately required strabismus surgery [9]. While these findings suggest that the modality of device use, specifically viewing distance, may be an important risk factor, the study's retrospective nature and reliance on self-reported data limit definitive causal conclusions.

### Time-trend and registry studies: suggestive associations, but strong confounding

A 12-year institutional longitudinal series identified a progressive increase in AACE incidence; notably, excessive smartphone usage was documented in a substantial proportion of patients and was statistically associated with steeper annual increases [11]. Although informative, such time-trend analyses are inherently ecological and cannot effectively disentangle smartphone exposure from concurrent secular shifts, including escalating educational intensity, sleep curtailment, psychosocial stressors, indoorization, and the pandemic surge in near-work demand. Furthermore, temporal shifts in the definition and ascertainment of excessive use may introduce measurement drift, potentially confounding longitudinal comparisons.

A multicenter Japanese registry analysis of AACE in patients aged 5–35 years demonstrates that device-use patterns vary significantly by age group; notably, pediatric patients reported lower device usage than older cohorts despite exhibiting larger angles of deviation [14]. These findings provide a necessary counterpoint to an exclusively device-centered etiology, suggesting that high device exposure is not a universal prerequisite and that age-related or patient-specific susceptibility significantly modulates the clinical phenotype.

## Exposure characteristics: smartphone viewing distance is often close and variable

A central premise in the smartphone-associated AACE discourse is that handheld digital displays are utilized at significantly shorter viewing distances than printed books or desktop monitors, thereby escalating accommodative and vergence demands. Empirical evidence corroborates this proximity while simultaneously highlighting substantial inter-individual variability. In a controlled experiment involving young adults reading from a smartphone for 60 minutes, the mean viewing distance was 29.2 ± 7.3 cm, with a temporal trend toward further shortening; notably, closer viewing correlated with increased asthenopic symptoms [17]. This quantitative observation is important: although the mean viewing distance approximates 30 cm, the standard deviation indicates a significant “tail” of users engaging at extreme proximity. In this review, we define extreme proximity as viewing distances of less than 20 cm, at which accommodative and vergence demands exceed 5 D and 5-meter angles, respectively. This aligns with clinical intuition suggesting that a specific subset of individuals, the high-demand users, adopts viewing behaviors that place excessive stress on the binocular vision system.

Motion-capture studies measuring head-to-hand distance report a wide range of smartphone viewing distances: approximately 13–33 cm when seated and 10–21 cm in the supine position, underscoring a posture-dependent reduction in working distance [18]. These values contrast sharply with conventional computer-viewing distances, which are typically 50–70 cm. Thus, smartphones and tablets likely impose significantly greater vergence demands than desktop monitors, not only due to viewing duration but also because of shorter, more variable working distances. Importantly, many retrospective reports fail to clarify whether myopic patients used devices with full correction, partial correction, or without spectacles. This uncertainty is clinically relevant, as uncorrected or undercorrected myopia reduces accommodative demand during near-viewing, thereby diminishing AC/A-driven vergence input. Across broader demographics, viewing distance and preferred character size vary systematically with refractive status and accommodative reserves, suggesting that individuals employ comfort-seeking strategies that actively modulate their visual exposure levels [19]. Smaller font sizes may encourage closer viewing, whereas larger font sizes may allow a longer viewing distance and thereby reduce accommodative and vergence demand [20]. Crucially, screen time and working distance represent distinct exposure metrics. Two individuals with identical daily usage durations may experience markedly different vergence and accommodative loads depending on viewing distance, posture, font size, and task-specific engagement (e.g., reading versus high-intensity gaming or video consumption). Therefore, the interpretation of retrospective exposure data, particularly self-reported screen time and viewing distance, is limited by a lack of granularity regarding device type, font size, and platform-specific usage patterns.

## Bias, confounding, and the “smartphone as a convenient villain”

### Publication and attention bias

The temporal proximity between smartphone use and AACE presents a compelling narrative; consequently, such cases are more likely to be documented, peer-reviewed, and cited compared to those lacking an identifiable modern trigger. This classic publication bias can artificially amplify the perceived strength of a novel association, particularly in the nascent stages of literature when the evidence base is dominated by case reports and small clinical series.

### Exposure misclassification and recall bias

The majority of existing studies rely on retrospective self-reported metrics, such as daily usage hours or subjective excessive use. Following the onset of diplopia, patients and their families may retrospectively seek causal explanations, leading to an overestimation of prior device exposure. Simultaneously, clinicians may probe smartphone usage history more rigorously due to growing interest in this potential cause. Such differential recall and interviewer bias can artificially inflate the observed associations, potentially misclassifying incidental exposure as a primary etiologic factor.

To establish a more definitive causal link, future research must transition from subjective self-reports to objective digital phenotyping. Integrating smartphone-based distance sensors and application programming interface (API) logging for screen time [21] will be helpful for quantifying actual visual loads and identifying true risk thresholds for AACE.

### Selection bias and referral effects

Tertiary referral centers may disproportionately encounter acute or dramatic clinical presentations, such as sudden-onset diplopia in students following prolonged smartphone use. This referral bias is likely amplified by the alarming nature of such presentations in adolescents and young adults, which often prompts both immediate referral to tertiary centers and the publication of case reports when a newly recognized contemporary exposure is identified. This phenomenon may have been compounded during periods of heightened public and clinical awareness, such as the shift to remote learning during the COVID-19 pandemic or the implementation of Japan’s GIGA School Program, which provided one tablet per student in public elementary and junior high schools by March 2021 [22]. Consequently, observed increases in case frequency may, at least in part, reflect shifts in healthcare-seeking behavior and referral patterns rather than a true rise in population-based incidence.

### Confounding by refractive error, near-work intensity, and lifestyle clustering

Smartphone exposure frequently clusters with other variables that plausibly impair binocular control, including prolonged near-work duration (independent of device type), reduced outdoor activity, sleep disturbances, academic pressure, and the rising prevalence of myopia. While large-scale studies suggest a link between device exposure and myopic risk, proving direct causality remains methodologically complex [23]. If the escalating prevalence of myopia, or its associated refractive correction, increases susceptibility to AACE, the observed association between smartphone use and AACE may be mediated by these shared determinants, forming a confounding cluster. Current evidence cannot definitively exclude the possibility that smartphone use serves primarily as a surrogate marker for a broader behavioral phenotype, characterized by intensive near-work loads and disrupted circadian rhythms. In susceptible individuals, however, smartphone use may interact with refractive status, limited binocular reserves, and near-viewing behaviors to precipitate AACE.

## Refractive error and uncorrected myopia: a cautious interpretation

Clinical reports frequently document cases among myopic patients, and longitudinal analyses indicate a more pronounced increase in incidence within this demographic [8, 11]. However, these findings do not necessarily establish a causal relationship between myopia and smartphone-associated AACE; rather, the observed association may reflect the increasing prevalence of myopia in younger generations, greater exposure to intensive near work, specific device-use behaviors, or differences in healthcare-seeking patterns. A recent Chinese case-control study identifies near work in a supine position as an independent risk factor for AACE, suggesting that posture-related near-work behavior may be clinically significant [13]. This finding is consistent with ergonomic data showing that supine smartphone use can shorten viewing distance [18].

A prevalent clinical hypothesis suggests that myopia may predispose individuals to near viewing distances that are excessively short, potentially attenuating blur cues and facilitating sustained, high vergence demand. While physiologically plausible, this remains unverified. Crucially, existing studies often lack standardized metrics regarding habitual correction (full vs. undercorrection), accommodative function, baseline heterophoria, or fusional reserves prior to the onset of esotropia. Consequently, refractive error should be regarded as a potential confounder, effect modifier, or surrogate marker, rather than a definitive causal factor, pending the emergence of robust prospective evidence.

## Mechanistic considerations: accommodation–vergence control and fusional “lock”

### Schor’s model as a conceptual scaffold

**Fig. 1:**
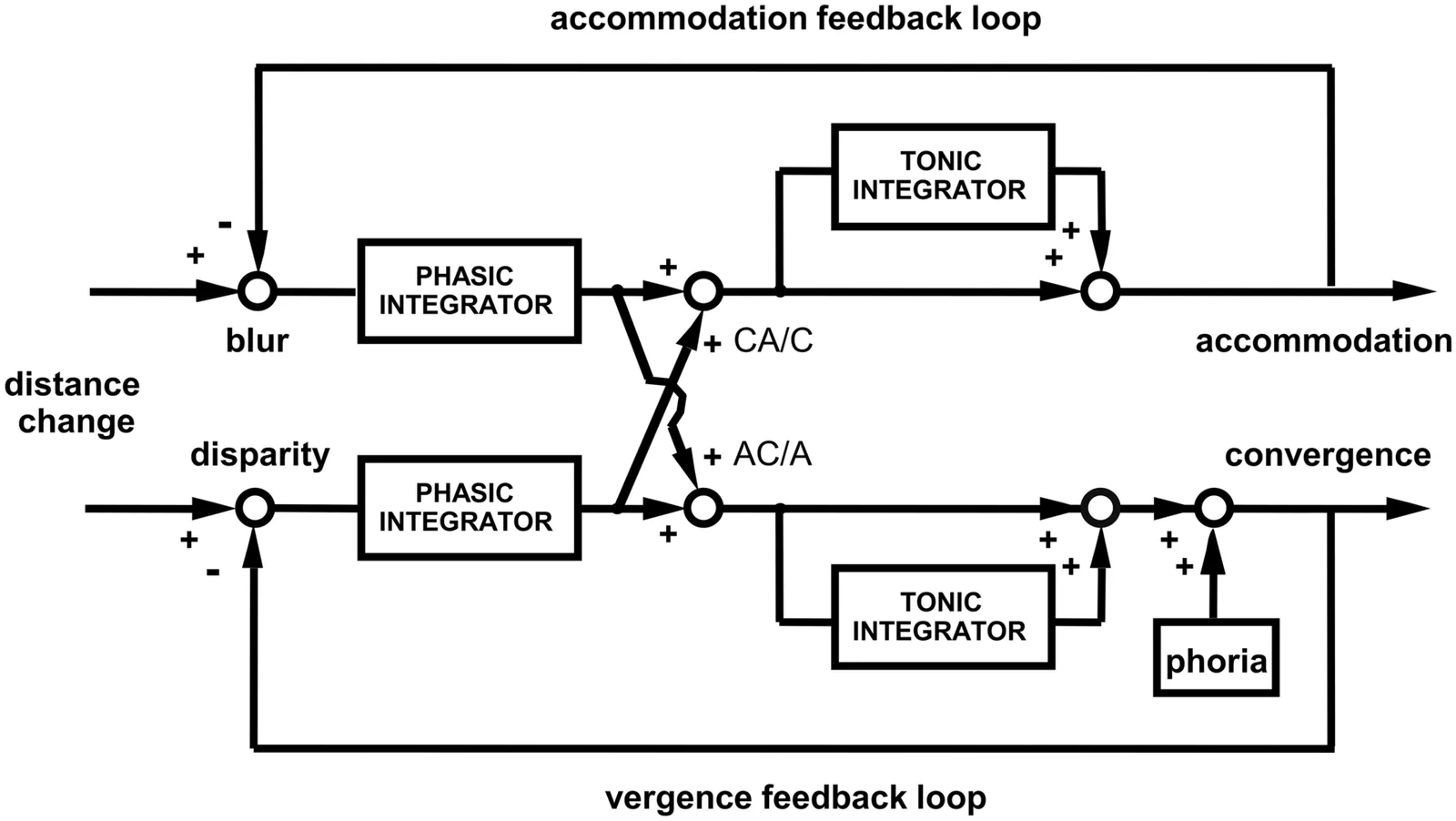
Schor’s model of accommodation and vergence and their interaction [24, 25]

The Schor model (Fig. 1) describes the accommodation and vergence control systems and the interactions between them. It predicts that, during sustained near work, adaptive changes occur in the tonic integrators of both systems, thereby relieving the continuous load on the phasic integrators via the feedback loops [24, 25].

According to this model, prolonged smartphone use at short viewing distances shifts the tonic vergence system toward a more convergent state and reduces the buffer between habitual visual demand and fusional capacity. Once fixation shifts to distance after intensive smartphone use, an esotropia with uncrossed diplopia may emerge. Under these hypothetical conditions, the vergence control system enters an open-loop state. In individuals with normal tonic integrator function, the esodeviation gradually returns to baseline, and binocular single vision is regained within several minutes. In contrast, in individuals with underlying subnormal binocular function, such as pre-existing microstrabismus or latent esophoria, distance esotropia may persist for a prolonged period.

Consistent with this framework, a Chinese case-control study identifies close-up work without spectacles as an independent risk factor for AACE [13], while a Japanese multicenter registry study [14] highlights uncorrected myopia as a significant risk factor for smartphone-associated AACE. When intensive near work is performed without myopia correction, the reduced accommodative demand diminishes neural input via the AC/A linkage (Fig. 1), thereby increasing the load on the tonic vergence integrator and accelerating vergence adaptation. As a result, the medial rectus muscles may maintain sustained contraction even during distance fixation until this adaptation resolves.

Experimental evidence [26] suggests that such sustained extraocular muscle contraction may increase intramuscular sarcomere density, potentially inducing structural shortening through muscle-length adaptation [27, 28]. Unlike transient neurological vergence adaptation, these structural alterations are relatively irreversible and may facilitate the transition to manifest AACE, even in individuals without prior binocular dysfunction. Although these physiological adaptations provide a plausible framework for understanding AACE in the context of intense near work, they remain speculative in the absence of direct longitudinal evidence in humans.

### The significance of small displays and limited peripheral visual cues: a speculative but principled hypothesis

One plausible hypothesis is that smartphones may exert a more pronounced impact on motor fusion dynamics than printed media or personal computers. This may be attributed to the restricted visual angle subtended by smartphone displays—typically approximately ±6° × ±12° at a 33 cm viewing distance—which limits the peripheral binocular cues essential for motor fusion. This effect is likely exacerbated in low-ambient light, where the background is perceptually suppressed or blurred. Experimental evidence underscores that fusional vergence ranges are highly dependent on stimulus size and retinal eccentricity, with a broad binocular context contributing significantly to vergence stability [29, 30]. Consequently, the narrowing of the functional field of view during smartphone use may compromise the fusion lock, thereby facilitating the decompensation from heterophoria to manifest heterotropia. Although direct evidence linking restricted peripheral input to AACE has yet to be reported, this mechanism provides a plausible physiological framework that warrants further clinical investigation.

## Clinical implications: what is reasonable to recommend now

Given the limited certainty regarding causal inference, balanced against the physiological plausibility of near-demand mechanisms, it is reasonable for clinicians to recommend pragmatic visual hygiene for patients presenting with recent-onset comitant esotropia or uncrossed diplopia, particularly in those with intensive handheld digital display use. This low-risk intervention is supported by evidence from nonrandomized studies, which suggest partial symptomatic improvement following a reduction in device usage and an increase in viewing distance [7, 15]. Recommended measures include maintaining a viewing distance of at least 30 cm, taking regular breaks during sustained near work, increasing font size to encourage greater viewing distances, avoiding handheld display use in the supine position, and ensuring optimal refractive correction. Such guidance may also be beneficial for symptomatic pre-teens and adolescents with asthenopia or intermittent diplopia, potentially mitigating the risk of progression to a fixed esotropia.

However, clinicians should avoid overstating the certainty of this association. Within a rigorous scientific framework, intensive smartphone or handheld digital-display use is most defensibly characterized as a postulated precipitating factor for AACE in specific patient subsets, provided that the substantial influence of confounding variables and bias is explicitly acknowledged [3, 9, 11, 15].

A comprehensive ophthalmic and neurologic evaluation is mandatory to rule out secondary etiologies. Assessment should precisely characterize binocular function and refractive status, ideally via cycloplegic refraction, and include an evaluation of ductions, versions, and ocular alignment at both near and distance. Furthermore, careful optic disc examination should be performed. Neuroimaging should be prioritized if neurologic deficits, optic disc edema, abduction limitations, or other red flags are identified.

## Conclusions

Prolonged handheld digital-display use, particularly when accompanied by short viewing distances, is associated with AACE in the current literature, although clinical improvement following reduced exposure is only sometimes observed. The evidence base remains limited by low-certainty study designs, and a robust population-level causal relationship has not yet been established. Handheld digital displays may act as precipitating factors within a multifactorial framework that includes screen time, ergonomic factors, refractive status, and psychosocial influences. Establishing definitive causality will require prospective, longitudinal studies that utilize objective exposure metrics and rigorous adjustment for potential confounders.
